# Melamine contamination in nutritional supplements - Is it an alarm bell for the general consumer, athletes, and ‘Weekend Warriors’?

**DOI:** 10.1186/s12937-015-0055-7

**Published:** 2015-07-17

**Authors:** Gary Gabriels, Mike Lambert, Pete Smith, Lubbe Wiesner, Donavon Hiss

**Affiliations:** 1 Division of Clinical Pharmacology, Department of Medicine, University of Cape Town, Faculty of Health Sciences, Observatory, Cape Town, 7925 South Africa; 2 Division of Exercise Science and Sports Medicine, Department of Human Biology, University of Cape Town, Cape Town, South Africa; 3 Department of Medical BioSciences, University of the Western Cape, Cape Town, South Africa

**Keywords:** Nutritional supplements, Information labels and warnings, Uric acid, Cyanuric acid, Pesticides, Protein, Laboratory screen testing

## Abstract

**Background:**

Nutritional supplements are used or experimented with by consumers, notably these are; competitive and recreational athletes of all ages, and ‘weekend warriors’. As a consequence the supplement industry has grown to meet the increasing demand. A Global Industry Analysts Inc. report indicates that the herbal supplement market has not declined during the worldwide recession, but in fact exhibited steady growth over the period 2008 to 2009. It is anticipated that the market will reach US$93.15 billion by the year 2015. These supplements may contain adulterated substances that may potentially have harmful short - and long-term health consequences to the consumer. “Scrap Melamine” is such an example, which has been implicated in the kidney failure and death of several cats, dogs and pigs. In China in 2008, reports described very severe health effects in infants and young children. At the time over 294 000 infants were screened and diagnosed with urinary tract stones and sand-like calculi associated with melamine in milk products, of which 50 000 infants were hospitalised, and at least six associated deaths, recorded. The extent that melamine contamination occurs in nutritional supplements is not known. Therefore, the aim of this study was to determine whether commercially available nutritional and traditional supplement products contain melamine, even though they are not declared by the manufacturer on the product label.

**Methods:**

A total of 138 nutritional supplements products were obtained from (i) direct purchases from shops, pharmacies and outlets, (ii) directly from consumers, and (iii) from suppliers, manufacturers and distributors. The products were laboratory analysed for melamine, using Tandem Liquid Chromatography Mass Spectrometry.

**Results:**

Forty-seven % of all the products (n = 138) tested positive for melamine. Eight-two % of the South African produced products (n = 27) tested positive and 58 % of the products imported into South Africa (n = 50) tested positive. The median concentration estimate for melamine in the products tested were, 6.0 μg/g for the 138 supplements tested, 8.9 μg/g for South African produced products, and 6.9 μg/g for products imported into South Africa.

**Conclusion:**

The melamine (*undeclared on product label*) levels detected in the nutritional supplements products investigated were within the Tolerable Daily intake (TDI) limit guidelines of 200 μg/g as set by WHO and others. Melamine over exposure within the context of the nutritional supplements consumption in the products investigated should not be of concern to the consumer provided the recommended guidelines of daily product use are adhered to. Further investigation is warranted to determine, (*i)* the link of melamine as *(part)* substitute for the perceived total declared protein content on the product label, (*ii*) cyanuric and uric acid presence in the supplement products that could form chemical-complex formation with melamine and/or analogues that could cause adverse health effects.

## Background

Nutritional supplements are used in particular by competitive and recreational athletes of all ages, and ‘weekend warriors’. The situation is further exacerbated by the general pressure placed on certain groups to use supplements. For example, young sports participants who are engaged in developmental and competitive phases of sport, encounter peer pressure to use supplements. As a consequence the supplement industry has grown to meet the increasing demands. A Global Industry Analysts Inc. report indicates that the supplement market has not declined during the worldwide recession, but in fact exhibited steady growth over the period 2008 to 2009. It is anticipated that the market will reach US$93.15 billion by the year 2015. These supplements may contain adulterated substances that may potentially have harmful short - and long-term health consequences to the consumer [1]. Also, insufficiencies in regulation of the supplement industry increase the risk of the nutritional supplements being contaminated. “Scrap Melamine” is an example of a food additive/adulterant contaminant. This is composed of multi-amine and has been used as a source of non-protein nitrogen, which falsely increases the claimed protein content [2–4]. Other sources and entry to humans have been via melamine resin migration to food [5], carry over from the illegal use of melamine in animal feed or feed ingredients, and then from feed to products of animal origin [5].

A variety of effects mentioned or associated with melamine intake, although inconclusive in humans, are, nephrolithiasis, chronic kidney inflammation, and bladder carcinoma [6–9]. As such, melamine has been implicated in the kidney failure and or disease, and deaths of several cats, dogs and pigs and the healthfulness of animals due to long-term exposure of maximum melamine baseline and adulteration levels [2, 10, 11]. Humans have a risk to urolithiasis formation following long-term exposure to low levels of melamine [12]. The pathogenic mechanism emanating via oxidative stress from increase free radical production that cause inflammation [6]. Furthermore, it is speculated from studies in rats that renal failure occurs as result of crystallization of ‘melamine’ in the kidney, leading to tubular cell necrosis, regeneration, and expansion [4, 7]. The mechanism of melamine-related urinary stones formation is still unclear [9].

Melamine also causes sperm cell abnormality, without any observed evidence of genotoxicity in prokaryotic or eukaryotic cells [2]. In humans toxicity has been reported although more specificity dose-dependent reproductive toxicity needs to be documented [2]. Melamine is excreted predominantly in the urine and hence essentially not metabolized in the human body although there are detectable amounts of melamine in tissue such as liver, spleen, bladder and brain [2, 8]. Developing organs in fetus *in utero* and neonates *ex utero* are highly susceptible to the melamine accumulation [8].

The China Melamine Crisis of 2008 reports described very severe health effects in infants and young children due to adulterated infant formula with melamine. At the time, over 294 000 infants were screened and diagnosed with uncommon in children, urinary tract stones and sand-like calculi associated with melamine in milk products, of which 50 000 infants were hospitalized, and at least six associated deaths, recorded [8, 13]. This situation may have been compounded due to the frequent intake of baby formula in early infancy, and may not provide enough time for the total clearance of the melamine between meals, causing gradual accumulation of the contaminant inside the body [8].

Whilst the infant formula melamine scandal has since subsided, there is evidence that melamine is ubiquitously present in the environment and the majority of the general population, including children, are most likely still exposed [14]. Due to the urgent response needed in the face of the melamine crisis, analytical methodology able to accurately determine this compound at trace levels became a requirement to identify potential health risks involved with consumption of contaminated foods consumed by humans [15]. Therefore the aim of this study was to determine whether commercially available nutritional and traditional supplement products contain melamine (1,3,5-triazine-2,4,6-triamine), even though the manufacturer does not declare them on the product label. A secondary aim was to compare the content to the Tolerable Daily Intake (TDI) of melamine.

## Methods

The nutritional supplements products assessed (n = 138) were obtained from (i) direct purchases from shops, pharmacies and outlets, (ii) directly from consumers, and (iii) from suppliers, manufacturers and distributors. The produced and purchased in South Africa products, where based on a combination of, (i) popular nutritional supplements brands acquired by a questionnaire survey from elite athletes, (ii) popular product sales as determined by information as acquired by shops, pharmacies and outlets which sold nutritional supplements, and (iii) budget constraints for the research project.

The nutritional supplement formulation types consisted of powder (P) 39 % (54), tablet ground to powder (P/T) 30 % (42), powder in capsule (P/C) 22 % (30), liquid in capsule (L/C) 6 % (8), liquid (L) 3 % (4). The products were laboratory analysed for melamine using Tandem Liquid Chromatography Mass Spectrometry. This laboratory technique was selected because it offers the desired multiple chemical compound (drug) separation, selectivity, and sensitivity with appropriate detection capability. The approach was to optimize (via infusion) the analytical instrumentation to detect on the Applied Biosystems Sciex API 3200 mass spectrometer for melamine. The chromatography method utilized prior to MS/MS detection was performed using an Agilent 1200 series HPLC with a Phenomenex Gemini C18 NX (5 μm, 110A, 50 x 2 mm) analytical column. The mobile phase consisted of acetonitrile: 0.1 % formic acid (1:1, v/v) and was delivered at a constant flow rate of 0.5 ml/min. The column was kept in a column compartment at 35 °C. The retention time for melamine was 0.26 minutes. For the analysis for melamine the transition from 127.1 to 68.1 atomic mass units was used. Calibration curve was established using prepared serial dilution to establish concentrations starting from 1000 ng/ml to 1.953 ng/ml (n = 3). For the assessment screen the Lower Limit of Quantification was 1.95 ng/ml (19 ng/g). The quadratic regression y = −0.00424X^2^ + 18.3X + 256 with correlation coefficient of 0.99 was used to estimate qualitative the concentration of melamine in the nutritional supplement products investigated.

## Results

Of the overall products acquired 47 % tested positive for melamine. The summarized data (Table 1) shows that a greater percentage of products (82 %) produced and purchased in South Africa, compared to those nutritional supplements products (58 %) imported and purchased in South Africa were contaminated with melamine. Based on median concentration values the supplement produced and purchased in South Africa had a higher level of melamine contamination (8860 ng/g) compared to those imported and purchased in South Africa (6940 ng/g).

**Table 1:** Summary of Nutritional Supplement products investigated for Melamine

		Overall samples acquired in South Africa	Produced and purchased in South Africa	Imported and purchased in South Africa
		(n = 138)	(n = 27)	(n = 50)
Qualitative Assessment Screen	Total No.	64	22	29
	Positives (%)	47	82	58
Estimate Concentration (*)	≥ LLOQ (ng/g) Low	218	513	218
	≥ LLOQ (ng/g) High	127030	127030	76419
	Median (ng/g)	6031	8860	6940

* 1ppm = 1mg/kg = 1000ng/g = 1 μg/g

Table 2 shows that of overall samples investigated, 38 % of products that had melamine contamination were powder products. Powder products (48 %) that were contaminated with melamine were highest in imported products purchased in South Africa. Products produced and purchased in South Africa having melamine contamination were highest (41 %) in tablet formulation.

**Table 2:** Nutritional Supplement formulation types investigated that tested ‘positive’ for Melamine contamination

Formulation Types	Number of ‘positives’ for Melamine (Samples acquired in South Africa)	Overall ‘positives’ for Melamine (%)	Produced and purchased in South Africa (n = 27)	Produced and purchased in South Africa (%)	Imported product purchased in South Africa n = 50	Imported product purchased in South Africa (%)
P	24	38	5	23	14	48
P/T	17	26	9	41	6	21
P/C	16	25	6	28	9	31
L/C	3	5	1	4	0	0
L	4	6	1	4	0	0
Total	64	100	17	100	29	100

Table 3 sets out the daily tolerable daily intake (TDI) for melamine as stipulated by respective agencies. TDI refers to the daily amount of a chemical that has been assessed safe for human being on long-term basis (usually whole lifetime).Table 4 presents global examples of melamine contamination in selected products [16].

**Table 3:** Tolerable Daily Intake (TDI) for Melamine

Organization	TDI Maximum Dose	Comment
WHO	0.2 mg/kg (2008)	Co-occurrence of melamine with cyanuric acid seems to be more toxic. Cyanuric acid 1,5 mg/kg (TDI).
	50 kg person (10 mg)	
	Infant Formula - 1ppm	
	Foods - 2.5 ppm	
EFSA	0.2 mg/kg (2010)	In line with WHO (2008)
	0.25 mg/kg (before 2010)	
FDA	2.5 ppm (2008)	“Acceptable” level – despite very few studies on melamine and human consumption
CAC	Infant Formula – 1mg/kg	United Nations Food Standards body
	Food and animal feed – 2.5 mg/kg	
SA	2.5 ppm	In line with WHO (2008)

World Health Organization (WHO), European Food Safety Authority (EFSA), Food and Drug Administration, South Africa, Codex Alimentarius Commission (CAC). http://en.wikipedia.org/wiki/Tolerable_daily_intake. (Accessed on the 10th December 2014)

**Table 4:** Global examples of Melamine contaminated levels

Product	mg/kg	Product	mg/kg
Animal Feed	3.3 – 21,000	Liquid milk	8.6
Beverage (coffee/orange juice	2	Contaminated Foods	0.38 – 945
Powdered milk products	1 – 6.196	Processed Food and Ingredients	0.6 – 6.694
Powdered infant formula	0.1 – 2.563	Whole eggs	2.9 – 4.7

http://www.who.int/foodsafety/areas_work/chemical-risks/melamine/en/ (Accessed on the 10th December 2014)

Based on average weight of a South African being 65.7 kg, http://en.wikipedia.org/wiki/Body_weight (accessed on the 22nd October 2014), Table 5 provides and illustrative example of the required amount to achieve the TDI limit as presented in Table 3.

**Table 5:** Illustrative examples of melamine contamination levels and consumption relative to accepted TDI

Description	High	Medium	Low
Concentration of Melamine (ng/g)	127030	6003	218
Formulation Type	P/C	P	P
Indication of Protein/amino acids content on Label (Y/N)	No	Yes	Yes
Product consumption quantity per day based on consumed and dose information (grams)	0.593	32	43
Indication of Melamine content on label (Y/N)	No	No	No
Local/ International Product purchase South Africa	Local	International	International
Dose information per tablet/capsule (grams)	0.593	-	-
Total Melamine consumption per day (mg)	0.075	0.192	0.009

Table 6 provides a synopsis for the melamine ‘positives’ products that were produced and purchased, or imported and purchased, in South Africa that were investigated for container label information, in the respective predetermine categories of potential dangers and warnings, scientific indication, disclaimers and claims *(Some products provided information in multiple categories)*.

**Table 6:** Pre-determine product label information for the Melamine ‘positives’ investigated

Information category	Total number of Melamine ‘positive’ products (n = 64)	Percentage of total ‘positives’ (%)	Highest number per information category for a given product investigated
Dangers and warnings	20	31	10
Scientific indication	20	31	21
Disclaimers	19	30	4
Claims	21	33	12

## Discussion

The study shows (Table 1) that of the overall samples acquired in South Africa (n = 138), 64 (47 %) products tested ‘positive’ for melamine as contaminant. For those products purchased and produced in South Africa the number of ‘positives’ for melamine (n = 27) was 22 (82 %), and imported products and purchased in South Africa (n = 50) 29 (58 %). None of the products identified as melamine ‘positive’ had information on the label stating that melamine may be present as contaminant. This misleading advertising may cause consequence for the health of the consumers due to chemical risk if non-approved ingredients are used or contamination occurs as pointed out by Venter et al. and Meltzer et al. [17, 18], and melamine adulteration used as ‘protein essence’, to falsely increase the protein content in products [5].

Many studies have been done in ‘animals’ and scientific assessment to directly link the state of disease *(various stage of disease)* to melamine contamination [6, 11, 19, 20]. The theory is that melamine forms the central nidus of the stone in the presence of acidified urine, causing hyperuraturia that may precipitate the acute urate nephropathy [9]. The morphology of the crystal suggests it may be a composite crystal in humans [7, 10, 13, 19]. From work done by Rai et al. [2] it is also known that melamine is not metabolized and has a very short half-life (approximately 3 h) in the plasma, and remains predominantly unchanged in urine. Melamine’s presence in the urine may therefore be an indicator of consumption of adulterated ‘food’ [2], hence several international agencies are working together on strategies to monitor and control melamine contamination [11]. Another source of adulteration could be via pesticide residues such as cryomazine in products that culminate in melamine as chemical product.

This finding of low level of melamine in products can be interpreted as indicating that there should be no concern for potential adverse health events, based on melamine content only and the TDI. However other co-determinants, co-contaminants [8] such as uric and cyanuric acid, not investigated in this study, may lead to precipitation of melamine chemical-complex [21]. Animals studies further suggest that the combination of melamine and cyanuric acid which is almost undissolvable in water [8], appeared to be more toxic than each substance alone [7, 19], and may therefore be a concern for human consumption. Liu et al. [9] also suggest that most proteins bind to melamine crystals [9]. The proteins may be derived from the consequences of cell injury, metabolic processes and binding functions [9]. These respective complexes may thus be contributory factors in the earlier stage of possible renal/kidney adverse condition or event occurring [13], which could play an important role in stone formation [9]. Melamine or melamine-complex accumulation may therefore occur due to consumption of products that leads to ‘depot formation’ in the kidney and other tissue. This may contribute to the cause of renal/ kidney impairment *‘injury’* over time. This indicates that melamine contaminated products (low levels) could contribute to adverse events, even though the prescribed dosage is adhered to within the TDI [4, 13]. The TDI value has seen a downward trend over time [22], which is indicative of cautious concern by international monitoring agencies, and may not be a desired indicator of consumption safety based on other factors and not on melamine only.

It follows that melamine contaminated nutritional supplements (*low levels*) may be the contributory tipping point for an adverse health event to occur. This could be due the composition of the consumers overall diet, that collectively may contain levels of melamine that allows the TDI to be exceeded. Evidence of this although not absolutely conclusive, *(has also not been shown in humans)*, is the significant presence of melamine residues *(contaminant)* in milk from dairy cattle for at least 6 days after the diet that contained the contaminant was removed [4]. Further, a study by Wu et al. [12]^,^ suggested that exposure to low- dose melamine-related products may have the potential to develop both uric acid and calcium urolithiasis in adults and children [12, 14].

There is a need for actual dosage consumption studies at extended and prescribed doses in both healthy and chronic kidney disease subjects to measure the retention capacity for melamine and /or melamine- complex in these respective environments. It is also important to further investigate the risk associated with dietary co-exposure, long-term exposure, and bacterial metabolism of melamine, cyanuric and related triazines, and the subsequent consequence of acute kidney injury, including crystal formation [9, 11, 21], particularly if these chemical moieties form part of nutritional supplement products.

## Limitations of study

That analysis for melamine ‘only’ as done in this nutritional supplement study is not adequate to show a possible product causal relationship for melamine crystal formation. The presence of uric acid and/or cyanuric acid, protein content, pesticide assessment (concentration) should be assessed in the same sample as they may be the contributory compounds for precipitation of the melamine-complex that may form the mechanism of kidney failure/ nephrotoxicity, and/or urialisis.

## Recommendations

Laboratory investigation needs to be done in nutritional supplement products for; (i) uric acid (ii) cyanuric acid, (iii) multiple protein assay systems to assess for adulteration [5, 23], and (iv) assessment for pesticides (cyromazine) [5, 21] that may convert to melamine, as by- or- end products. These findings would contribute to a better understanding of the precipitation mechanism of the product-complex, which would lead to accumulation in the kidney, and compromise renal function integrity [14]. To enhance the overall quality of supplements there needs to be an ongoing concerted effort to improve regulatory frameworks [24, 25] and methods of surveillance and vigilance for undeclared chemical compounds in nutritional supplement products. Further, governments should have the responsibility to equip appropriately, the law enforcement capabilities, that will alert timeously via ‘early warning consumer communication system’ of ‘problem products’. Also with the necessary rigor have these products recalled, to avoid a nutritional supplement scandal, as was the case with the baby formula milk products in 2008. In the absence and evaluation of controlled human clinical trials, and improving clinical diagnosis of adverse health effects, that may be associated with consumption of dietary/nutritional, patients and doctors’ reports, and case studies should be (re)assessed. Supplements products should therefore be scientifically tested, to ensure that side- (adverse) effects are not inaccurately attributed and diagnosed, and that may further lead to the establishment of incorrect policies, and/or warnings of nutritional supplement products. To determine the extent/and demography of kidney disease, and reflect on the clinical records to determine possible association of the kidney disease and burden of disease, due to melamine and related chemical-complex formation.

## Conclusion

The number of ‘positive’ tested products for melamine in the overall cohort was 47 % (64), for South African produced products 82 % (22), and, for imported products, bought in South Africa 58 % (58). The median concentration estimate for melamine in the products were, 6.0 μg/g for the overall cohort, 8.9 μg/g for South African produced products, and 6.9 μg/g for imported products, bought in South Africa (Tables 1, 2 and 3). Whilst the concentration levels of melamine detected in the products is low overall *(as contaminant),* the number of products containing the contaminant should place nutritional supplement products under greater scrutiny. The melamine *(undeclared on product label)* levels detected in the nutritional supplements products investigated were within the TDI limit guidelines of 0.2 mg/kg as set by WHO and other organisations. So melamine over-exposure should not be of concern to the consumer, provided, the recommended guidelines of daily product use, are adhered to. Further, the WHO TDI may not be an appropriate guideline limit as it, (i) overlooks the effect of chronic dosing, (ii) that consumption of melamine contamination could come from different nutritional supplement products sources, collectively or consecutively, and (iii) that accumulation of low levels of melamine as contaminant or in the presence of other chemical- complex formation, over time, could lead to a health hazard or incident.

Therefore, cautious monitoring intermittently, by health safety authorities for melamine as contaminant in nutritional supplement products would be adequate at this juncture.

The findings further suggest research investigation pertaining to melamine, in relation to actual dose consumption as current findings were based on the assumption that consumers would have adherence to the prescribed label dosage information, rather than substantially exceeding this amount as may be the case. The potential link of melamine (complex) to adverse health consequences such as renal dysfunction will need to be tested by assessing nutritional supplement products for uric and cyanuric acid, pesticides, specifically cryomazine, and assessing for melamine adulteration via multiple respective protein assays systems.

In conclusion, reforming the nutritional supplement sector should continually consist of; (i) problem or potential problem identification, (ii) benchmarking the way towards greater consumption safety for the consumer, (iii) refined, meaningful and implementable regulation, and, (iv) adequate well trained officials, controls and enforcement.
